# Fungal Prosthetic Joint Infection in Revised Knee Arthroplasty: An Orthopaedic Surgeon’s Nightmare

**DOI:** 10.3390/diagnostics12071606

**Published:** 2022-06-30

**Authors:** Christos Koutserimpas, Symeon Naoum, Kalliopi Alpantaki, Konstantinos Raptis, Konstantinos Dretakis, Georgia Vrioni, George Samonis

**Affiliations:** 1 Department of Orthopaedics and Traumatology, “251” Hellenic Air Force General Hospital of Athens, 11525 Athens, Greece; chrisku91@hotmail.com (C.K.); naoumsimeon@gmail.com (S.N.); kraptis1981@hotmail.gr (K.R.); 2 Department of Orthopaedics and Traumatology, “Venizeleion” General Hospital of Heraklion, 714-09 Crete, Greece; apopaki@yahoo.gr; 3 2nd Department of Orthopaedics, “Hygeia” General Hospital of Athens, 151-23 Marousi, Greece; kostasdretakis@gmail.com; 4 Department of Microbiology, Medical School, National and Kapodistrian University of Athens, 115-27 Athens, Greece; gvrioni@med.uoa.gr; 5 Department of Medicine, University of Crete, 71500 Heraklion, Greece; 6 First Department of Medical Oncology, “Metropolitan” Hospital, Neon Faliron, 185-47 Attica, Greece

**Keywords:** knee infection, *Candida* knee infection, *Aspergillus* knee infection, fungal osteoarticular infection

## Abstract

Fungal prosthetic joint infections (PJIs), despite the fact that they are rare, represent a devastating complication. Such infections in revised knee arthroplasties pose a unique surgical and medical challenge. A rare case of *Candida parapsilosis* PJI in revised knee arthroplasty is reported. Furthermore, a thorough review of all published fungal PJIs cases in revised knee arthroplasties is provided. A 72-year-old female with total knee replacement surgery due to osteoarthritis 10 years ago, followed by two revision surgeries six and two years ago due to aseptic loosening, presented with signs and symptoms of septic loosening of the knee components. Resection arthroplasty and cement-spacer placement was performed and periprosthetic tissue cultures yielded *Candida parapsilosis*. The patient was commenced on proper antifungal treatment (AFT) for six months and then the second stage of the revision surgery was performed successfully. From 2000 to 2022, a total of 46 patients with median age 69 years [interquartile range (IQR = 10)], suffering fungal PJI occurring in revised knee arthroplasty have been reported. The median time from initial arthroplasty to symptoms’ onset was 12 months (IQR = 14). Cultures of local material (52.2%) and histology (6.5%) were the reported diagnostic method, while *Candida* species were the most commonly isolated fungi. Regarding surgical management, two-stage revision arthroplasty (TSRA) was performed in most cases (54.3%), with median time-interval of six months (IQR = 6) between the two stages. Regarding AFT, fluconazole was the preferred antifungal compound (78.3%), followed by voriconazole and amphotericin B (19.6% each). The median duration of AFT was five months (IQR = 4.5). Infection’s outcome was successful in 38 cases (82.6%). Fungal PJIs, especially in revised knee arthroplasties, are devastating complications. A combination of AFT and TSRA seems to be the treatment of choice. TSRA in these cases poses a special challenge, since major bone defects may be present. Therapeutic procedures remain unclear, thus additional research is needed.

## 1. Introduction

Total knee arthroplasty (TKA) represents one of the most commonly performed orthopedic procedures worldwide. Osteoarthritis, which is the main cause for TKA, limits the joint movement and affects millions of patients [1]. TKA improves quality of life, minimizing pain and restoring joint movement [2]. Joint reconstruction surgery has evolved throughout the years, encompassing minimally invasive surgical approaches, perioperative pain management and blood transfusion reduction protocols, and navigation or robotic systems, as well as new prosthetic materials [2,3,4,5].

The number of primary TKAs doubled from 1991 to 2010 in USA, while it is estimated to increase even more by 2050 [6]. Due to the large number of patients undergoing TKA, as well as the expansion of life-expectancy, the need for revision reconstruction surgeries has also been increased [7]. The main causes of prosthetic failure include infection, aseptic loosening, and periprosthetic fractures [5,6,7].

Revision surgery is a very demanding procedure, generating outcomes inferior to those of primary knee arthroplasty and with higher risk of complications [7]. The surgeons have to handle various and different technical challenges regarding surgical exposure and approach, bone loss management as well as the appropriate implant selection [7].

Prosthetic joint infections (PJIs), occurring in 0.7–2% of all cases, despite the fact that they are rare, represent a devastating complication with major consequences, as far as the quality of the patient’s life is concerned, while, in some cases, they may be proven fatal [8]. Early PJI cases most commonly occur due to intraoperative contamination. Delayed and late presentations are usually characterized by progressive, persistent pain, and are almost always hematogenous in origin [7,8,9]. Regarding the risk factors for PJI, operative time, tourniquet time, cement type, diabetes, obesity, American Society of Anesthesiology (ASA) score, and blood transfusion requirement have been reported [9].

Furthermore, given the increased incidence of fungal infections due to aging population, as well as to increasing number of immunosuppressed patients, fungi have been held responsible in about 1–2% of PJIs [10,11,12].

Fungal pathogens are usually found in immunocompromised hosts, suffering immunosuppression and other host-dependent factors such as diabetes or prolonged antibiotic therapy, as well as multiple surgeries for PJIs [10,11,12].

Due to the rarity of these infections, no clear guidelines exist regarding management, while currently, on the basis of limited data, a two-stage revision arthroplasty (TSRA) combined with prolonged antifungal treatment (AFT) is suggested [10,13]. As far as PJIs in revised knee arthroplasties is concerned, re-revision surgery may be extremely demanding, dealing with huge osseous deficits leading often to very poor surgical outcomes. However, fungal PJIs in revised knee arthroplasties have not yet been studied separately, even though they pose a unique surgical and medical challenge.

The present study reports a rare case of *Candida parapsilosis* PJI in revised knee arthroplasty. The case is relatively rare, since only 16 other cases have been reported so far in the literature. Furthermore, this study, by reviewing all published fungal PJIs cases in revised TKA, makes an effort to clarify both medical and the surgical treatment options and their effectiveness, in order to conclude to best management.

## 2. Case Presentation

A 72-year-old female with body mass index of 33 kg/m^2^ presented to the orthopaedic out-patient clinic due to knee pain, swelling, and inability to bear full weight, starting at least four months ago. The patient was afebrile, while her medical history was remarkable for a total knee replacement surgery due to osteoarthritis 10 years ago, followed by two revision surgeries six and two years ago due to aseptic loosening. In all three procedures, the patient had prophylactic vancomycin for 48 h following surgery. Furthermore, she had a history of diabetes mellitus (DM), hypertension, and heart failure.

**Figure 1:**
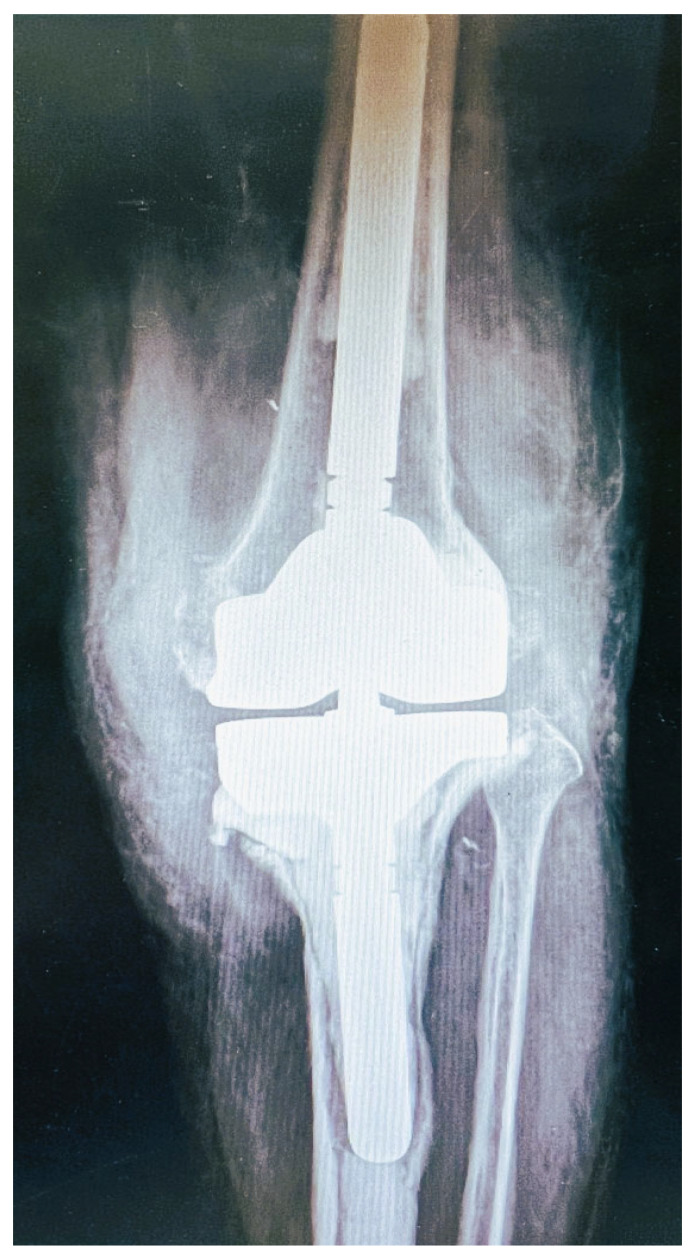
Pre-operative X-ray: anteroposterior view of the revised knee arthroplasty. Loosening is evident especially at the tibia component.

X-ray of the knee exhibited loosening of the prosthetic joint, especially in the tibia component (Figure 1). Laboratory examination showed C-reactive protein (CRP) = 52.7 mg/L and erythrocyte sedimentation rate (ESR) = 64 mm/1st h. She underwent resection arthroplasty surgery the following day. The knee prosthetic joint, as well as the cement were thoroughly removed. Surgical debridement was performed and the bone sequestrum was also removed. Finally, cement-spacer with vancomycin and gentamycin was placed. The patient received empirically antimicrobial intravenous treatment with vancomycin.

Cultures from the peri-prosthetic tissues, as well as from the removed implants yielded the same *Candida parapsilosis*, as well as a methicillin-resistant *S. epidermitis*, while blood cultures were negative. The fungal isolate was susceptible to amphotericin B (0.125 μg/mL), fluconazole (<3 μg/mL), posaconazole (0.094 μg/mL), itraconazole (<0.5 μg/mL), voriconazole (0.047 μg/mL), caspofungin (2 μg/mL), and micafungin (<0.04 μg/mL).

As soon as the results of the cultures were available, she was commenced on intravenous micafungin and continued vancomycin for six weeks. She was then discharged, on oral voriconazole and moxifloxacin for a total of six and three months, respectively. The laboratory examination, at six months after discharge, revealed normal values of CRP (6.4 mg/L) and ESR (18 mm/1st h), while at that point in time she had no signs or symptoms of a knee PJI and all the rest routine laboratory tests were with normal limits.

**Figure 2:**
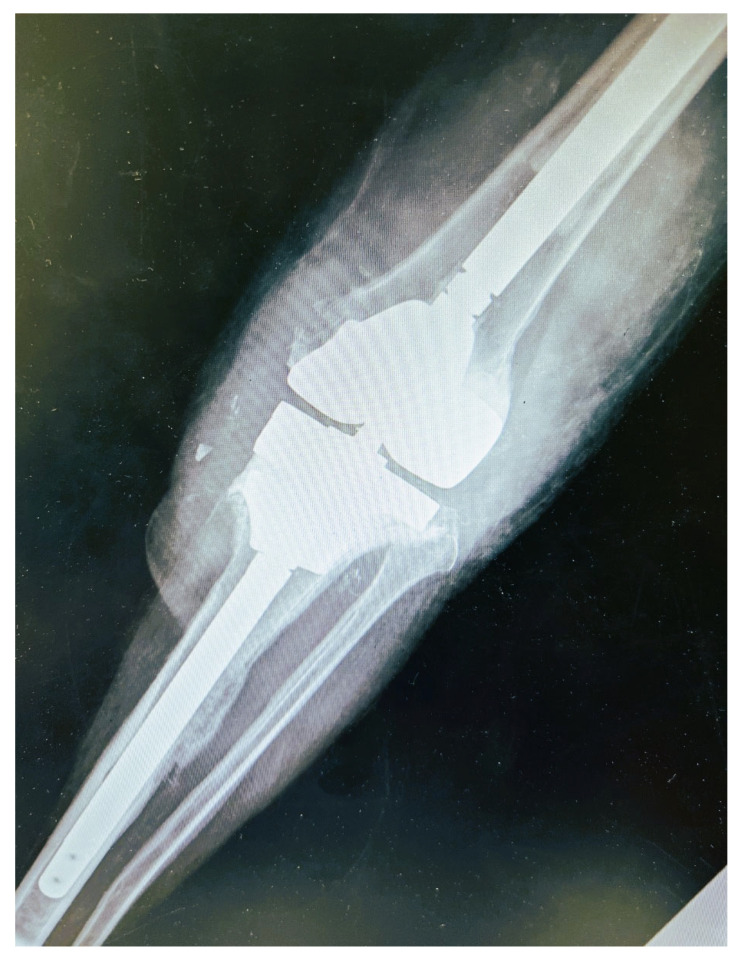
Post-operative anteroposterior X-ray view. A constrained prosthesis with long femoral and tibia stems was placed. A tantalum porous scaffold was placed in the proximal tibia due to bone loss, while augments supported the femoral and tibia components.

The second stage of the revision surgery was performed six months after the initial resection arthroplasty and spacer cement placement. A constrained prosthesis with long femoral and tibia stems was placed. A tantalum porous scaffold was placed in the proximal tibia due to bone loss, while augments supported the femoral and tibia components due to osseous deficits (Figure 2). The patient had an uneventful recovery. Two years following the final reconstruction joint surgery, the patient is fine with no signs or symptoms of PJI, while flexion of the knee has reached 90 degrees.

## 3. Discussion

Fungal PJIs are quite uncommon and particularly challenging as far as management is concerned [10,11,14,15]. Most such infections are caused by *Candida* species, followed by other fungi [10,11,12]. The risk of PJI is higher for knee arthroplasty than hip arthroplasty, which may be attributed to the larger mobility of knee joint and soft tissue, as well as to the lesser soft tissue coverage [8,9]. It is of paramount importance that information regarding AFT agents, AFT duration and its success rate, as well as the kind of surgical procedures, the use of antifungal agents in cement, and the time intervals between the two stages of TSRA be clarified, so that the best applicable of both medical and surgical management of these patients may be provided.

The presented case exhibited the surgical and medical challenges that such fungal infections encompass in revised arthroplasties. Non-*albicans Candida* PJI are rare, and, thus, should be reported for better understanding treatment options and outcomes. The patient, suffering PJI in a revised knee arthroplasty due to *Candida parapsilosis*, was successfully treated with causative AFT and two-stage revision arthroplasty (TSRA) separated with a time-interval of six months. It seems that fungal PJI in revised knee arthroplasties could be considered a separate clinical entity, with increased morbidity, demanding proper diagnostic and therapeutic management.

A meticulous electronic search of the PubMed, MEDLINE, and EMBASE databases was also performed to identify all existing articles regarding cases of fungal periprosthetic joint infections occurring in revised knee arthroplasties. Alone and/or in combination, the terms “fungal”, “fungal infection”, “mold knee infection”, “periprosthetic joint infection”, “total knee replacement infection”, “total knee arthroplasty infection”, “revision knee surgery”, “*Candida* periprosthetic joint infection”, “*Aspergillus* joint infection”, “Coccidioidal joint infection”, “*Acremonium* joint infection”, “*Alternaria* joint infection”, “*Histoplasma* joint infection”, “*Syncephalastrum* joint infection”, and “*Phialemonium* joint infection” were searched. Following the identification of these cases, individual references listed in each publication were further investigated for ascertainment of additional cases. The present review included infections from yeasts and mold since the surgical management remains the same for both cases. However, the microorganism has been identified, the patient receives the appropriate treatment either for yeast or mold.

The present review was limited to papers published from January 2000 to April 2022, in English and in peer-reviewed journals. Expert opinions, book chapters, studies on animals, on cadavers or in-vitro investigations, as well as abstracts in scientific meetings were excluded.

The data extracted from these studies included age, gender, the presence of immunosuppressive condition, the presence of co-infection, C-reactive protein levels, erythrocyte sedimentation rate, time interval from joint implantation to symptom onset and from symptoms’ onset to diagnosis, number of previous revisions of the affected arthroplasty, reason of previous revision(s), duration, and type of anti-fungal treatment (AFT) as well as the type of surgical intervention. Furthermore, the results of medical and surgical treatment, along with the follow-up of each case, were recorded and evaluated. Treatment was considered successful if all signs and symptoms of the infection had disappeared and no recurrence was observed during the follow-up period.

Table 1 summarizes the main characteristics of all reported cases of fungal PJI in revised knee arthroplasty. A total of 46 patients (17; 37% males) suffering fungal PJI occurring in revised knee arthroplasty, covering a 22-year period, were identified [16,17,18,19,20,21,22,23,24,25,26,27,28,29,30,31,32,33,34,35,36]. The studied population’s median age was 69 years [range = 27–84, interquartile range (IQR) = 10].

The median time from initial arthroplasty implantation surgery to symptoms’ onset was 12 months (range = 1–72, IQR = 14), while the median time from symptoms’ onset to culture-confirmed diagnosis was two months (range = 1.1–6, IQR = 2.75).

Detailed information regarding immunosuppressive conditions, as well as symptomatology are presented in Table 1. It is of note that 17 patients (37%) were suffering at least one immunosuppressive condition, according to the available information from each report.

Fungal PJIs incidence is expected to increase, due to the expanding rates of prosthetic joint reconstruction [8,35,36]. Both immunosuppression and systemic diseases have been commonly recognized as risk factors for invasive fungal infections [8,23,28]. In the present review, a total of 17 patients (37%) were immunocompromised.

Fungal PJIs occur most often hematogenously [12,17]. Nevertheless, intraoperative contamination by skin fungi could also happen. The median time interval, in all published fungal PJIs in revision knee arthroplasties, between initial joint reconstruction surgery and onset of symptoms was 12 months, indicative of hematogenous spread, based on the fact that early-onset infection is usually acquired during implantation. In these cases, the median time from the symptoms’ onset to definite diagnosis was two months. Fungal PJIs typically present with mild symptomatology and, therefore, diagnosis may be delayed [12,17]. Among these cases, the indolent course of *Candida* PJIs may be indicative of direct contamination [37].

Regarding the causative fungal organisms, the most frequently isolated one was *Candida parapsilosis*, reported in 16 cases (34.8%), followed by *C. albicans* in 15 (32.6%), *C. glabrata* in four (8.7%), *Aspergillus* spp. in three (6.5%), while *C. tropicalis*, *C. freyschussii*, *C. lusitanae*, *Acremonium* spp., *Alternaria* spp., *Histoplasma capsulatum*, *Phialemonium curvatum*, *Syncephalastrum racemosum* had caused one case each (2.2%). Co-infection was present in 13 cases (28.3%), with the most common microorganism being *Staphylococcus* spp. (9 cases; 69.2%), followed by *Streptococcus* spp. (3; 23%), while Gram-positive bacteria, *Mycobacterium*, *Corynebacterium* group, *Pseudomonas aeruginosa* were also cultured once each (1; 7.7%).

It is of note that concomitant bacterial infection has been reported in the literature in about 15–20% of fungal PJIs [38]. These cases refer to revised arthroplasties and surgically re-explored knee joints; thus, this could have contributed to the higher prevalence of bacteria isolation.

The median number of prior revisions of the affected knee prosthetic joint was 1 (range = 1–4, IQR = 1), while the most common reason for revision surgery was infection (22 cases; 47.8%), periprosthetic fracture (3; 6.5%) and aseptic loosening (1; 7.7%), while in 20 cases the reasons of previous revisions were not reported.

Table 2 highlights diagnostic techniques, including imaging indicating the infection, as well as the methods of firm diagnosis. Regarding imaging methods, plain X-ray or CT scan were performed in nine patients (19.6%), followed by bone scan in four (8.7%), while magnetic resonance imaging (MRI) was not implemented in any case.

Regarding imaging studies, they may be quite valuable but typically radiographic imaging does not provide a definitive diagnosis [8,39]. In general, plain radiographs should be obtained in the beginning of a suspected PJI, so that a prosthetic loosening and/or fracture may be observed. However, they lack high sensitivity and specificity for definite PJI diagnosis [39].

Definite diagnosis was possible through cultures and/or histopathology. Moreover, in 44 cases (95.7%), fungal species were cultured. In three cases, fungal PJI was diagnosed through histopathology (6.5%), while serology testing was not reported in any case. In particular, in case no. 18, fungal PJI was diagnosed through both histopathology and cultures.

If PJI is suspected, initial diagnostic algorithm consists of plain radiography and measurement of serum inflammatory markers (e.g., ESR and CRP) [39]. Thereafter, a diagnostic arthrocentesis may be implemented, unless there is clinical evidence of PJI (e.g., sinus tract) and surgical debridement should take place [8,40]. Regarding intraoperative specimens, at least three periprosthetic tissue samples (ideally five) should be obtained with different instruments in order to guarantee any absence of cross-contamination between specimens. The samples should be sent for culture, as well as histological examination. Fungal cultures should be performed in patients with chronic or refractory infection, as well as in immunosuppressed hosts [28,40].

Table 3 summarizes surgical and AFT options, duration of treatment and infection’s outcome. Regarding surgical management, two-stage revision arthroplasty (TSRA) was performed in most cases (25; 54.3%), with a median time-interval of six months (range = 2–20, IQR = 6) between the two stages, followed by one-stage revision arthroplasty (OSRA) (8; 17.4%), resection arthroplasty (RA) (5; 10.9%), arthrodesis (3; 6.5%), and debridement (3; 6.5%), while two cases did not receive any surgical treatment (4.3%).

Although clear guidelines regarding treatment of fungal PJIs do not exist, it seems that TSRA represents the preferred surgical management [10,11,13]. Other surgical interventions include debridement and retention of prosthesis, OSRA, resection arthroplasty with no reimplantation or amputation [8,10,11,16,18,39]. It should be noted that amputation and arthrodesis may drastically diminish the patient’s quality of life, while OSRA has doubtful results in bacterial PJIs [11]. Nevertheless, it must be kept in mind that in cases of re-revision joint surgery, these decisions should not be made lightly, since these operations are extremely demanding. It is of note that TSRA’s success rate was 92%, while ORSA, RA arthrodesis, and debridement exhibited success rates of 75%, 80%, 67%, and 67%, respectively (Table 3).

Regarding AFT, 20 cases (43.5%) were treated with a single antifungal regimen, 20 (43.5%) with two, either simultaneously or consecutively, and five (10.9%) were treated with more than two antifungal agents, while in one case no data existed about AFT. The median duration of AFT was five months (range = 2–24, IQR = 4.5).

Fluconazole was the preferred agent in 36 cases ((78.3%), in 17 (47.2%) as monotherapy), followed by voriconazole in nine cases ((19.6%), in two (22.2%) as monotherapy), amphotericin B in nine ((19.6%), not as monotherapy), caspofungin, in five ((10.9%), not as monotherapy), flucytosine in four ((8.7%), not as monotherapy), and anidulafungin and micafungin in two each ((4.3%), none as monotherapy).

More specifically, regarding *Candida* PJIs, which are the most common ones (38 cases out of 46 (82.6%)), the preferred agent was fluconazole (30 cases (78.9%); 15 as monotherapy (50%)), followed by voriconazole (five cases (12.2%); none as monotherapy), caspofungin (five cases (12.2%); none as monotherapy), amphotericin B (four cases (10.5%); none as monotherapy), micafungin and anidulafungin (two cases each (5.3%); none as monotherapy). The majority of *C. albicans* isolates are susceptible to fluconazole and echinocandins. However, in case of fluconazole-resistant candida spp., such as *C. glabrata*, fluconazole should not be initially used. Echinocandins are the initial AFT agents of choice for osteoarticular infections due to *C. glabrata*, followed by step-down therapy with oral azoles based on the susceptibilities [12,17,25].

It is of note that currently, no guidelines exist for prophylactic AFT in high-risk, immunocompromised patients undergoing TKA. Nevertheless, taking into account that the number of immunocompromised hosts is increasing, along with the increased number of patients undergoing TKA and the rising of fungal infections, this should be the subject of future research.

The duration of treatment generally depends on the clinical and laboratory findings of each case as well as on physicians’ experience with such situations [10,36,40]. Attention should be paid in performing susceptibility testing in order that precise MIC values be acquired, following the isolation of the fungus, due to the fact that various species of fungi are featured by resistance to specific antifungal agents [11]. Regarding AFT, fluconazole was the preferred antifungal compound (78.3%), followed by voriconazole and amphotericin B (19.6% each). Fluconazole was extensively used in the published cases, despite its inefficacy against molds [41]. Nevertheless, it must be taken into consideration that both fluconazole and amphotericin B deoxycholate were the only AFT options in the early years of the studied cases [41,42]. Moreover, fluconazole has been associated with severe hepatotoxicity and, consequently, liver function should be monitored regularly during extended fluconazole treatment [41]. In addition, amphotericin B may be toxic (e.g., renal dysfunction), resulting in restricting its long-term use. On the other hand, liposomal compounds of amphotericin B have significantly diminished the drug’s nephrotoxicity [42,43]. Voriconazole was firstly introduced in 2003 and has been proven the drug of choice against *Aspergillus* spp. [43,44]. This compound has spectacularly transformed the management of *Aspergillus* infections in the last decades. This agent has all the features of azole agents, while being far less hepatotoxic and much less nephrotoxic than amphotericin compounds [44,45].

In 27 cases, antimicrobial or antifungal regimen in cement was used: a single agent in 13 cases (48.1%) and two agents in 14 (51.9%). Vancomycin was the preferred regimen in 16 cases ((59.3%), in three (18.8%) as single regimen), followed by amphotericin B in 10 ((37%), in eight (80%) as single regimen), gentamycin in six ((22.2%), not as single regimen), voriconazole in three ((11.1%), in two (66.6%) as monotherapy), meropenem in two ((7.4%), not as monotherapy) and tobramycin, piperacillin, ceftriaxone in one case each ((3.7%), none as monotherapy).

During the 2000–2022 study period, an infection’s outcome was successful in 38 cases (82.6%), while the mortality rate was (2.2%). It is notable, however, that the success rate drops to 75% in the cases of bacterial co-infection.

PJIs, along with other invasive fungal infections, represent a major cause of morbidity and mortality in current medical practice. Optimal treatment of fungal PJIs remains unclear since no certain guidelines exist regarding the antifungal regimen and the indicated surgical intervention. TSRA and long-term AFT are proposed due to lack of data. Publications about outcomes based on certain AFTs, its duration and its success rate, as well as the type of surgery, the use of antifungal agents in cement, and the time intervals between the two stages of TSRA, are of utmost importance for the clarification of the best medical treatment and the improvement of the surgical management of these cases. In the reported cases, TSRA was the preferred surgical intervention, while the time interval between the two-stages was six months.

Furthermore, it should be noted that re-revision joint surgeries are extremely demanding. Adequate treatment of bone defects poses a special challenge for orthopedic surgeons [46]. Management of bone defects, leading to stable and lasting support platform for the implantation materials, is of paramount importance for favorable outcomes. Additionally, it allows the correct alignment of the prosthetic and limb components, as well as restoring the height of the joint interline, which is essential for joint mobility. Several options exist for the management of bone defects in such cases, including bone cement with or without reinforcement with screws, modular metallic augmentations, impacted bone graft, structural homologous graft, and, more recently, metal metaphyseal cones, and metaphyseal sleeves [46,47]. In the reported patient, a tantalum porous scaffold was placed in the proximal tibia due to bone loss, while augments supported the femoral and tibia components. Porous tantalum has an interconnecting architecture and excellent osteoconductivity. The stress load is distributed homogeneously on porous tantalum implants, minimizing the possibility of periprosthetic dissolution and failure of the implant caused by stress shielding. Moreover, porous tantalum has high frictional coefficient against bone, enhancing early-stage stability as an implant, while it promotes osteogenesis and osteointegration [46].

## 4. Conclusions

Fungal PJIs, especially in revised knee arthroplasties, represent a challenge regarding diagnosis and management. A multidisciplinary approach is mandatory, since a combination of AFT and TSRA seems to be the treatment of choice. TSRA in these cases poses a special challenge since major bone defects may be present and thorough pre-operative planning is essential. Since the results of therapeutic procedures and policies remain unclear, additional information and research are needed, focusing on proper treatment and/or prophylaxis’ policies so that optimal management approach to be concluded.

## Figures and Tables

**Table 1:** Patients’ demographics, comorbidities, responsible fungus, affected joint, bacterial co-infection, time (T) interval from joint implantation to symptom onset and from symptom to diagnosis, number of previous revisions in the same joint, c-reactive protein, (CRP), and Erythrocyte Sedimentation Rate (ESR) at presentation. (-): Not mentioned in the original cases.

Case No	Year	Author	Country of Origin	Gender/Age	Fungus	Co-Infection	CRP mg/L	ESR mm/h	Immuno-Suppressive Medication and Conditions	Number of Previous Revisions	Reason of Previous Revision	T. from Implanta-tion to Symptom-Matology (Months)	T. from Symptoms Onset to Diagnosis (Months)
1	2018	Gao et al. [16]	China	F/52	*Acremonium* *strictum*	-	0.348	17	-	1	-	-	-
2	2018	Brown et al. [17]	USA	F/55	*Alternaria* spp.	-	35	36	-	Yes (NA)	Infection	-	-
3	2018	Gao et al. [16]	China	F/63	*Aspergillus* spp.	-	4.99	25	Diabetes Mellitus	3	-	-	-
4	2018	Gao et al. [16]	China	M/63	*Aspergillus* spp.	Gram positive bacteria, mycobacterium	10	92	-	2	-	-	-
5	2001	Baumann et al. [18]	USA	F/27	*Aspergillus fumigatus*	-	37	55	-	1	Aseptic loosening	53	0.1
6	2017	Nowbakht et al. [19]	USA	M/77	*Histoplasma capsulatum*	Group B *Streptococci*	-	-	-	3	periprosthetic fracture	8	0.3
7	2012	Anagnostakos et al. [20]	Germany	M/64	*Phialemonium curvatum*	-	>20	-	-	1	Infection	-	-
8	2002	Ceffa et al. [21]	Italy	F/72	*Syncephalastrum* *racemosum*	*Corynebacterium* group	-	-	-	1	Infection	2	2
9	2014	Klatte et al. [22]	Germany	M/69	*C. parapsilosis*	*S. epidermidis*	>22	-	Diabetes Mellitus, cancer, peripheral vascular disease, chronic obstructive lung disease	1	Infection	21	1
10	2014	Klatte et al. [22]	Germany	F/82	*C. parapsilosis*	-	>22	-	-	2	Periprosthetic fracture	3	2
11	2014	Klatte et al. [17]	Germany	M/74	*C. lusitaniae*	*S. aureus* *Strep. mitis*	>22	-	Myocardial infarction, chronic obstructive lung disease	4	Infection	12	5
12	2014	Klatte et al. [22]	Germany	M/46	*C. parapsilosis*	*Strep.* spec.	>22	-	-	2	Infection	17	6
13	2012	Hwang et al. [23]	Korea	F/66	*Rhodotorula* mucilaginosa	MRSA	29	71	Rheumatoid arthritis	1	Infection	13	-
14	2012	Hwang et al. [23]	Korea	F/65	*C. albicans*	-	64	32	-	1	Infection	15	-
15	2013	Ueng et al. [24]	Taiwan	F/84	*C. albicans*	-	-	-	-	1	-	2	1
16	2013	Ueng et al. [24]	Taiwan	F/64	*C. albicans*	-	-	-	Diabetes Mellitus	2	-	17	2.5
17	2000	Badrul and Ruslan [25]	Malaysia	M/64	*C. albicans*	-	-	-	-	1	Infection	1	1
18	2019	Keuning et al. [26]	Netherlands	F/72	*C. parapsilosis*	-	16	67	Rheumatoid arthritis, psoriasis	1	Infection	12	2
19	2002	Açikgöz et al. [27]	Turkey	F/70	*C. glabrata*	-	-	-	-	1	Infection	6	6
20	2003	Lerch et al. [28]	Germany	F/78	*C. albicans*	*S. aureus*	-	40	-	1	Infection	-	-
21	2017	Ji et al. [29]	China	M/72	*C. albicans*	-	-	-	Hypertension, Diabetes Mellitus, chronic bronchitis	2	Infection	13	-
22	2017	Ji et al. [29]	China	F/76	*C. glabrata*	-	-	-	Coronary heart disease	1	-	3	-
23	2017	Ji et al. [29]	China	F/49	*C. albicans*	-	-	-	-	1	-	7	-
24	2017	Ji et al. [29]	China	M/76	*C. albicans*	*S. lentus*	-	-	-	2	Infection	14	-
25	2017	Ji et al. [29]	China	F/77	*C. parapsilosis*		-	-	Hypertension, cancer, coronary heart disease	2	-	21	-
26	2017	Ji et al. [29]	China	F/69	*C. parapsilosis*	-	-	-	Hypertension, Diabetes Mellitus, chronic bronchitis	3	Infection	20	-
27	2018	Brown et al. [17]	USA	M/81	*C. albicans*	-	>35	>36	-	1	Infection	-	-
28	2018	Brown et al. [17]	USA	F/74	*C. parapsilosis*	-	>35	>36	-	1	Infection	-	-
29	2018	Brown et al. [17]	USA	F/56	*C. parapsilosis*	-	>35	>36	-	1	Infection	-	-
30	2018	Brown et al. [17]	USA	M/71	*C. parapsilosis*	-	>35	>36	-	1	Infection	-	-
31	2018	Brown et al. [17]	USA	M/70	*C. albicans*	-	>35	>36	-	1	Infection	-	-
32	2016	Jenny et al. [30]	France	F/53	*C. albicans*	-	-	-	-	1	Infection	-	-
33	2021	Mafrachi et al. [31]	Jordan	F/60	*C. parapsilosis*		127	78	-	1	Infection	3	-
34	2018	Gao et al. [16]	China	F/78	*C. parapsilosis*, *C. tropicalis*	-	29	0.3	-	3	-	12	-
35	2018	Gao et al. [16]	China	F/58	*C. freyschussii*	-	1	75	-	2	-	12	-
36	2018	Gao et al. [16]	China	M/64	*C. glabrata*	-	2	-	Coronary heart disease	2	-	14	-
37	2018	Gao et al. [16]	China	M/54	*C. parapsilosis*	*Staphylococcus*	7.49	25	-	2	-	5	-
38	2018	Gao et al. [16]	China	M/67	*C. parapsilosis*	-	0.48	20	-	2	-	5	-
39	2018	Gao et al. [16]	China	F/69	*C. albicans*	*S. cohnii*	-	-	Hypertension, Diabetes Mellitus	2	-	3	-
40	2018	Gao et al. [16]	China	M/66	*C. parapsilosis*	*S. epidermidis*, *Pseudomonas aeruginosa*	1.65	21	-	2	-	46	-
41	2010	Graw et al. [32]	USA	F/73	*C. albicans*	coagulase-negative *Staphylococcus*	2.6	-	Hypertension, obesity, atrial fibrillation, adenocarcinoma of the uterus	1	Periprosthetic Fracture	1	-
42	2005	Lejko-Zupanc et al. [33]	Slovenia	-/73	*C. glabrata*	-	-	-	-	1	-	72	-
43	2009	Bland and Thomas [34]	USA	F/55	*C. albicans*	-	-	-	Diabetes Mellitus, rheumatoid arthritis	1	-	2	-
44	2017	Cobo et al. [35]	Spain	M/66	*C. albicans*	-	-	-	Splenectomy	1	-	19	-
45	2018	Lee et al. [36]	Korea	F/71	*C. parapsilosis*				Diabetes Mellitus, hypertension, chronic kidney diseases	2	-	9	-
46	2018	Lee et al. [36]	Korea	F/71	*C. parapsilosis*	-	-	-	Diabetes Mellitus, hypertension, chronic kidney diseases	1	-	30	-

**Table 2:** Definite diagnosis of periprosthetic joint infections caused by fungus and imaging techniques that each case underwent during the process of diagnosing the infection, CT: computer tomography. (+): indicating that the method was used for diagnosis of the infection, (−): indicating that the method was not used for diagnosis of the infection.

Case	C/T X-ray	Bone Scanning with ^99m^Tc	Cultures	Biopsy
1	−	−	joint fluid, tissue specimen	-
2	−	−	joint fluid, tissue specimen	-
3	−	−	joint fluid, tissue specimen	-
4	−	−	joint fluid, tissue specimen	-
5	−	+	-	tissue specimen
6	+	−	tissue specimen	-
7	−	+	-	tissue specimen
8	−	−	tissue specimen	-
9	−	−	joint fluid, tissue specimen	-
10	−	−	joint fluid, tissue specimen	-
11	−	−	joint fluid, tissue specimen	-
12	−	−	joint fluid, tissue specimen	-
13	−	−	tissue specimen	-
14	−	−	tissue specimen	-
15	−	−	tissue specimen	-
16	−	−	tissue specimen	-
17	−	−	joint fluid	-
18	+	−	joint fluid	tissue specimen
19	+	−	joint fluid, tissue specimen	-
20	+	−	tissue specimen	-
21	−	−	joint fluid	-
22	−	−	joint fluid	-
23	−	−	joint fluid	-
24	−	−	joint fluid	-
25	−	−	joint fluid	-
26	−	−	joint fluid	-
27	−	−	joint fluid	-
28	−	−	joint fluid	-
29	−	−	joint fluid	-
30	−	−	joint fluid	-
31	−	−	joint fluid	-
32	−	−	tissue specimen	-
33	+	−	joint fluid, tissue specimen	-
34	−	−	joint fluid, tissue specimen	-
35	−	−	joint fluid, tissue specimen	-
36	−	−	joint fluid, tissue specimen	-
37	−	−	joint fluid, tissue specimen	-
38	−	−	joint fluid, tissue specimen	-
39	−	−	joint fluid, tissue specimen	-
40	−	−	joint fluid, tissue specimen	-
41	+	−	joint fluid, tissue specimen, bone specimen	-
42	−	−	tissue specimen	-
43	+	−	joint fluid, tissue specimen	-
44	−	−	joint fluid, tissue specimen	-
45	+	+	tissue specimen	-
46	+	+	tissue specimen	-

**Table 3:** Surgical and antifungal treatment, follow-up, and infection outcome of the reported cases. ST: Surgical Treatment, TSRA: two-stage revision arthroplasty, OSRA: one-stage revision arthroplasty, AFT: antifungal treatment, LS: lifelong suppression, NS: no surgery, RA: resection arthroplasty, NA: not available.

Case	ST	Time between Stages in TSRA (Months)	Antimicrobial Regimen in Cement	Antifungal Treatment (AFT)	Total Duration of AFT (Months)	Follow-Up (Months)	Outcome
1	TSRA	9	Voriconazole	Voriconazole, Fluconazole	6.5	30	Success
2	TSRA	6	Amphotericin B	NA	-	60	-
3	TSRA	7	-	Fluconazole	8.5	80	Success
4	TSRA (2x spacer exchange before final implantation)	14	-	Fluconazole	3	51	Failure
5	TSRA	3.5	-	Amphotericin B, Fluconazole	10.5	60	Success
6	TSRA	9	Voriconazole	Itraconazole	24	24	Success
7	OSRA	-	-	Voriconazole	6	5	Success
8	TSRA	2.5	-	Amphotericin B, Voriconazole	-	36	Success
9	OSRA	-	-	Flucytosin, Amphotericin B,	2	30	Failure
10	TSRA	20	-	Flucytosin Amphotericin B	2	30	Success
11	NS	-	-	Voriconazole	2	30	Success
12	NS	-	-	Flucytosin, Amphotericin B,	2	30	Success
13	TSRA	2.5	Vancomycin	Amphotericin B, Fluconazole	6	48	Success
14	Arthrodesis	-	Vancomycin	Amphotericin B, Fluconazole	6	48	Failure
15	TSRA	2	Vancomycin, Piperacillin	Fluconazole	>10	-	Success
16	RA	-	Vancomycin, Ceftriaxone	Fluconazole	>10	-	Failure (Death)
17	Debridement	-	-	Fluconazole	12	62	Failure
18	TSRA	3	Amphotericin B	Voriconazole, Micafungin	5	12	Success
19	Arthrodesis	-	-	Fluconazole	-	30	Success
20	Arthrodesis	-	-	Fosfomycin, Teicoplanin, Fluconazole	>2	-	Success
21	OSRA	-	Gentamicin, Vancomycin	Vancomycin, Fluconazole	>3	6	Success
22	OSRA	-	Gentamicin, Vancomycin	Vancomycin,	>3	6	Success
23	OSRA	-	Gentamicin, Vancomycin	Fluconazole	>3	6	Success
24	OSRA	-	Gentamicin, Vancomycin	Vancomycin, Fluconazole	>3	6	Success
25	OSRA	-	Gentamicin, Vancomycin	Fluconazole	>3	6	Failure
26	OSRA	-	Gentamicin, Vancomycin	Vancomycin, Fluconazole	>3	6	Success
27	TSRA	NA	Amphotericin B	Fluconazole	-	48	Success
28	TSRA	NA	Amphotericin B	Fluconazole	-	48	Success
29	TSRA	NA	Amphotericin B	Fluconazole	-	48	Success
30	TSRA	NA	Amphotericin B	Fluconazole	-	48	Success
31	TSRA	NA	Amphotericin B	Fluconazole	-	48	Success
32	Debridement	-	-	Caspofungine, Voriconazole, Flucytosine	2	24	Success
33	TSRA	3	Amphotericin B	Capsofungin, Fluconazole	15	8	Success
34	TSRA	3	Vancomycin, Meropenem	Fluconazole	7.5	80	Success
35	TSRA	3	Vancomycin	Voriconazole, Fluconazole	7.5	74	Success
36	TSRA	9	Vancomycin, Meropenem	Fluconazole	5	129	Success
37	TSRA	10	Voriconazole, Vancomycin	Fluconazole, Voriconazole	6	32	Failure
38	TSRA	6	Amphotericin B, Vancomycin	Fluconazole	2.5	66	Success
39	TSRA	6	-	Fluconazole	4.5	25	Success
40	TSRA	13	Amphotericin B, Vancomycin	Fluconazole, Rifampicin	5	64	Success
41	TSRA	9	Tobramycin, Vancomycin	Fluconazole, Vancomycin, Voriconazole, Caspofungin, Daptomycin	7	24	Success
42	RA	-	-	Amphotericin B, Fluconazole, Caspofungin	-	36	Success
43	RA	-	-	Amphotericin B, Micafungin, Fluconazole	6	-	Success
44	Debridement	-	-	Caspofungin, Fluconazole	6.5	3	Success
45	RA	-	-	Fluconazole, Anidulafungin	21	32	Success
46	RA	-	-	Fluconazole, Anidulafungin	2	48	Success (Death of unrelated disease

## Data Availability

Not applicable.
